# Isolated and combined association of excessive screen time and physical inactivity with negative self-rated health in adolescents

**DOI:** 10.1590/1984-0462/2023/41/2022077

**Published:** 2023-04-07

**Authors:** Jean Carlos Parmigiani de Marco, Fernanda Ulsula de Souza, André de Araújo Pinto, Mateus Augusto Bim, Rita Maria dos Santos Puga Barbosa, Markus Vinicius Nahas, Andreia Pelegrini

**Affiliations:** a Universidade do Estado de Santa Catarina, Florianópolis, SC, Brazil.; b Universidade Estadual de Roraima, Boa Vista, RR, Brazil.; c Universidade Federal do Amazonas (UFAM), Manaus, AM, Brazil.; d Universidade Federal de Santa Catarina, Florianópolis, SC, Brazil.

**Keywords:** Diagnostic self evaluation, Adolescents, Physical activity, Epidemiology, Risk factors, Autoavaliação diagnóstica, Adolescentes, Atividade física, Epidemiologia, Fatores de risco

## Abstract

**Objective:**

The aim of this study was to analyze isolated and combined associations of physical inactivity excessive screen time with negative self-rated health, according to sex, among school adolescents.

**Methods:**

In this cross-sectional study conducted with 2,517 adolescents in Amazonas State, participants were asked about their self-rated health with the following question: How do you rate your health? Responses were dichotomized into positive (excellent and good) and negative (regular, bad, and terrible). Information on sex, age group, family income, physical activity, and screen time (watching TV, using a computer, or playing video games) was collected through a self-administered questionnaire. Adolescents simultaneously classified as physically inactive (<60 min/day) and having excessive screen time (>2 h/day) were considered to have two risk factors. Data was analyzed using binary logistic regression.

**Results:**

Out of every 10 adolescents, 2 had a negative self-rated health. After adjusting for age and family income, there were no isolated or combined associations between physical inactivity or excessive screen time and negative self-rated health in girls. In boys, negative self-rated health was associated with insufficient levels of physical activity (odds ratio [OR]: 2.39; 95% confidence interval [CI]: 1.03–5.59) and with the accumulation of two risk factors (OR: 1.61; 95%CI 1.10–2.34).

**Conclusions:**

Being insufficiently active and the combination of physical inactivity and excessive screen time become exposure factors to the negative self-rated health of adolescent boys.

## RESUMO

**Objetivo:**

Analisar a associação isolada e combinada do tempo excessivo de tela e inatividade física com a autopercepção negativa de saúde, de acordo com o sexo, em adolescentes estudantes.

**Métodos:**

Estudo transversal conduzido em 2.517 adolescentes amazonenses, os quais foram questionados sobre a autopercepção de saúde: “Como você considera a sua saúde?”, dicotomizada em positiva (excelente, boa) e negativa (regular, ruim, péssima). Informações sobre sexo, faixa etária, renda familiar, atividade física e tempo excessivo de tela (assistindo TV, usando o computador ou jogando *videogame*) foram coletadas mediante questionário autoadministrado. Aqueles classificados, simultaneamente, como fisicamente inativos (<60 min/dia) e com tempo excessivo de tela (>2 horas/dia) foram considerados com dois fatores de risco. Os dados foram analisados utilizando-se a regressão logística binária.

**Resultados:**

Dois em cada dez adolescentes apresentaram autopercepção negativa de saúde. Após o ajuste pelas variáveis idade e renda familiar, não foram observadas, no sexo feminino, associações da inatividade física e do tempo excessivo de tela, de maneira isolada ou agrupada, com a autopercepção negativa de saúde. No sexo masculino, a percepção negativa de saúde foi associada com os níveis insuficientes de atividade física (*odds ratio* — OR: 2,39; intervalo de confiança — IC95% 1,03–5,59) e com o acúmulo de dois fatores de risco (OR: 1,61; IC95% 1,10–2,34).

**Conclusões:**

Ser insuficientemente ativo e associar a inatividade física com tempo excessivo de tela tornam-se fatores de exposição à percepção negativa em saúde de meninos adolescentes.

## INTRODUCTION

Self-rated health has been considered an important indicator of general health status^1^ as well as a strong predictor of mortality,^2^ despite being a multidimensional phenomenon.^1^ Adolescents, in particular, have been the focus of studies assessing health conditions worldwide because of the important physiological and anatomical transformations that occur during the typical maturation process in this phase of life.^3^ Furthermore, psychological changes are observed throughout adolescence, leading to behavioral changes that may generate internal, physical, emotional, and social conflicts.^4^

Within this context, evidence indicates a trend toward a reduction in physical activity levels^5^ and an increase in the time spent on screen time^6^ during adolescence. Given that physical inactivity^7^ and sedentary lifestyle^8^ are public health problems with high economic costs to society,^9,10^ it is necessary to promote changes in risk behaviors to heap benefits on the general health of the population.^11^

In Brazil, data from the National Survey of School Health showed that most adolescents (13–17 years old) have a positive self-rated health (69.1%), with a higher prevalence in boys (78.2%) than in girls (60.4%). However, data also indicated a reduction in self-reported positive health with age among adolescents. In both sexes, there is a higher prevalence of positive self-rated health between 13 and 15 years of age (girls: 63.7%; boys: 79.9%) than between 16 and 17 years of age (girls: 54.4%; boys: 75%). Of note, positive self-rated health was found to be similar among adolescents enrolled in private (70.7%) and public (68.9%) schools.^12^

Considering the negative impacts that excessive screen time and physical inactivity provide^9,10^ and that self-rated health reflects on the individual’s general health condition,^1^ studies sought to analyze the effect that such risk behaviors had on self-perception of health.^1,13^ It is possible to observe that physically inactive^13^ and those who spend high total screen time^1^ have 2.31 and 1.25, respectively, a greater chance of having a negative self-rated health.

Knowledge of the prevalence of positive self-rated health in adolescents and a better understanding of the groups most likely to experience negative self-rated health can support healthcare managers, physical education teachers, and family members in designing strategies to promote health-related behavioral changes. Studies pointed out that the groups that most frequently report negative self-rated health are girls, adolescents whose parents have low education levels, adolescents with low economic levels, as well as those who practice less physical activity, are sedentary, or have inadequate nutritional status.^1,14,15^ Most studies addressing self-rated health and associated factors investigated physical activity and sedentary behavior independently.^1^ Furthermore, it is noted that boys and girls show differences when assessing their own health,^16^ demonstrating the need to assess the self-rated health according to the sex of adolescents. No study was found evaluating, either separately or in combination, the relationship of these two behavioral factors with self-rated health. Therefore, this study aimed to assess separate and combined associations of excessive screen time and physical inactivity with negative self-rated health, according to sex, among school adolescents.

## METHOD

This is an epidemiological, cross-sectional study with a school-based design, conducted as part of a research project carried out in 2011 in Amazonas State, Brazil, entitled “Lifestyle and health indicators of Amazonas high school students.” The research was approved by the Human Research Ethics Committee at the Federal University of Amazonas (protocol CAAE-0302.0.115.000-11).

The sample was composed of adolescents of both sexes, aged 14–19 years, enrolled in high schools belonging to the state public education network in five municipalities of Amazonas State, namely, Itacoatiara, Manaus, Parintins, Presidente Figueiredo, and São Gabriel da Cachoeira. These municipalities were intentionally selected for their accessibility, in contrast to the reality of most municipalities in Amazonas State, which are riverine and can only be accessed by boat.

According to information from the Amazonas State Secretariat for Education (SEDUC), a total of 88,562 adolescents were regularly enrolled in the five municipalities in 2011. In Manaus, the school selection process was composed of three stages:

1. Proportional stratification by education district (six districts);
2. Stratification by state public schools according to the number of students (large schools, 500 students or more; medium-sized schools, 201–499 students; and small schools, up to 200 students); and
3. Conglomerate selection by school shift, class, and year. All students present in class at the time of data collection were invited to participate in the research. Because Itacoatiara, Parintins, Presidente Figueiredo, and São Gabriel da Cachoeira had a small number of schools, selection in these municipalities consisted of stages (2) and (3) only. In Presidente Figueiredo, where there were only two schools, all adolescents were invited to participate in the study (census sampling).

For sample size calculation, we considered an estimated prevalence of 50% (unknown outcome), a confidence interval of 95%, a sampling error of 5% points, and a design effect of 1.5.^17^ An additional 10% was added to account for possible losses and refusals, resulting in a minimum sample size of 2,485 adolescents (Itacoatiara=580, Manaus=631, Parintins=587, Presidente Figueiredo=264, and São Gabriel da Cachoeira=423). Given the conglomerate nature of the sampling process, whereby all students present in class on the day of data collection were invited to participate, the number of adolescents included in the study exceeded the minimum sample size, totaling 2,517 adolescents.

SEDUC was asked to send an invitation to the selected schools, requesting their authorization for data collection. All principals of participating schools were informed about the importance and objectives of the research. Data collection was carried out by a team of trained researchers, on the days and times scheduled with principals and physical education teachers, who made their classes available for the application of questionnaires.

A team of trained researchers visited all selected classes to inform the adolescents about the research and its relevance. Students were officially invited to participate and received an informed consent form, which had to be signed by their parents or legal guardians (for students aged less than 18 years) or by themselves (for students aged 18 years and older). Adolescents who agreed to participate and provided the informed consent form were also asked to sign an assent form.

The data of the present study were obtained through the questionnaire “Comportamentos dos adolescentes catarinenses” (COMPAC) prepared on the basis of Global School-Based Student Health Survey, its face and content validity was previously tested on 117 adolescents, presenting reproducibility value of 0.51–0.96, being considered valid for application in adolescents from 14 to 19 years of age.^18^

The dependent variable was self-rated health, which was measured by the question “How do you rate your health?” Responses were grouped into negative self-perceptions (regular, bad, or terrible) and positive self-perceptions (excellent or good).

A questionnaire was applied to collect information on sociodemographic characteristics such as sex (male or female), age (14–18 years), and family income, as well as behavioral variables such as physical activity and screen time.

The family’s minimum wage reported in a previous Brazilian study^19^ was used for analysis of family income, categorized as follows: up to 2 minimum wages, 2–5 minimum wages, 6–10 minimum wages, and ≥11 minimum wages. The minimum wage was R$ 545 during the time of data collection (2011).

Physical activity was estimated from the weekly frequency (0–7 days a week) of moderate to vigorous physical activity multiplied by the daily practice time (I do not practice physical activity, <30 min a day, 30–59 min a day, or ≥60 min a day). Adolescents who performed less than 60 min of moderate or vigorous physical activity daily were classified as physically inactive and those who carried out 60 min or more were classified as physically active.^20^

Screen time was evaluated considering the time spent watching TV, using the computer, and/or playing video games during the week and weekend. Screen time was categorized into up to 2 h/day and more than 2 h/day, as prior recommended.^21^ For analysis purposes, adolescents classified simultaneously as physically inactive (<60 min/day) and having excessive screen time (>2 h/day) were considered to have two risk factors.

Data were initially analyzed using descriptive statistics (mean, standard deviation, and frequency distribution). To assess possible associations between physical activity and screen time, either separately and combined, with self-perception health, we stratified data by sex and then applied crude and adjusted binary logistic regression by age and family income. Analysis of the association between self-rated health and combined factors did not include adolescents with both healthy behaviors (physically active and ≤2 h of screen time). Odds ratios (ORs) and respective confidence intervals (CIs) were estimated. All analyses were performed at a significance level of 5% using the IBM SPSS Statistics software version 20.0.

## RESULTS

The total number of adolescents evaluated was 3,267. Of the total number of questionnaires collected, 382 were considered losses due to logistical problems during transport between cities, and another 368 were removed from the evaluation process due to incomplete responses in the questionnaire, totaling 2,517 adolescents eligible for the study.

Table 1 shows the general characteristics of adolescents in the total sample stratified by sex. All variables differed between sexes, with the exception of age and sedentary behavior. Of the 2,517 study participants, the majority were female (56.1%), aged between 16 and 17 years (56.1%), and had a monthly income of up to 2 minimum wages (64%). It was observed that 19.3% of adolescents have a negative self-rated health, 71.6% spend total screen time >2 h/day, 93% are insufficiently active, and 66.4% of adolescents presented a combination of excessive screen time and insufficient physical activity.

**Table 1.** General characteristics of the sample of adolescents. Amazonas, 2011.

	General sample n (%)	Male (n=1106) n (%)	Female (n=1411) n (%)	p-value
Age (years)				
14–15	519 (20.6)	220 (19.9)	299 (21.2)	0.188
16–17	1,413 (56.1)	610 (55.1)	803 (56.9)	
18–19	585 (23.2)	276 (25.0)	309 (21.9)	
Monthly income (salary)				
Up to 2 minimum wages	1,612 (64.0)	662 (59.9)	950 (67.3)	**<0.001**
3–5 minimum wages	710 (28.2)	333 (30.1)	377 (26.7)	
6 or more minimum wages	195 (7.8)	111 (10.0)	84 (6.0)	
Self-rated health				
Positive	2030 (80.7)	942 (85.2)	1088 (77.1)	**<0.001**
Negative	487 (19.3)	164 (14.8)	323 (22.9)	
Sedentary behavior (h)				
≤2	714 (28.4)	328 (29.7)	386 (27.4)	0.204
>2	1803 (71.6)	778 (70.3)	1025 (72.6)	
Physical activity				
Active	175 (7.0)	114 (10.3)	61 (4.3)	**<0.001**
Insufficiently active	2342 (93.0)	992 (89.7)	1350 (95.7)	
Combined factors				
Healthy	43 (1.7)	29 (2.6)	14 (1.0)	**<0.001**
1	803 (31.9)	384 (34.7)	419 (29.7)	
2	1671 (66.4)	693 (62.7)	978 (69.3)	

Bold indicates significant p values.

In males, it was observed that when the exposure factors were analyzed, either separately or in combination, there was only an association of the combined factors with the negative self-rated health in the crude analysis. When the final model was adjusted for age and income, it was observed that boys who were insufficiently active (OR: 2.39; 95%CI 1.03–5.59) and those who simultaneously accumulated two risk factors (OR: 1.61; 95%CI 1.10–2.34) were more likely to have a worse health perception (Table 2). No associations were observed between the variables investigated and the negative self-rated health in females.

**Table 2.** Isolated and combined association between excessive screen time and physical inactivity with negative self-rated health in adolescents, according to sex. Amazonas, 2011.

		Self-rated health	
		Crude OR (95%CI)	Adjusted OR (95%CI)*
Male	Screen time (h)		
	≤2	1	1
	>2	1.32 (0.90–1.92)	1.37 (0.92–2.04)
	Physical activity		
	Active	1	1
	Insufficiently active	1.91 (0.98–3.74)	2.39 (1.03–5.59)
	Combined factors		
	1	1	1
	2	1.61 (1.11–2.34)	1.61 (1.10–2.34)
Female	Screen time (h)		
	≤2	1	1
	>2	1.21 (0.91–1.62)	1.22 (0.91–1.64)
	Physical activity		
	Active	1	1
	Insufficiently active	1.00 (0.54–1.83)	1.28 (0.61–2.69)
	Combined factors		
	1	1	1
	2	1.28 (0.97–1.70)	1.24 (0.94–1.65)

OR: odds ratio; 95%CI: 95% confidence interval. *Adjusted for age and family income.

## DISCUSSION

In this study, it was possible to observe associations between negative self-rated health and physical inactivity in boys. When risk behaviors were grouped, we found that the chances of having a negative self-rated health were higher in boys with both risk factors than in boys with only one risk factor.

The prevalence of negative self-rated health in the present study was 19.3%, being higher in girls (22.9%) than in boys (14.8%). Similar prevalence rates were observed in studies conducted in Florianópolis, Santa Catarina State,^13^ and Belo Horizonte, Minas Gerais State.^14^ However, the prevalence rates found here were lower than those observed in Pernambuco (33% of girls and 19% of boys had negative self-rated health)^14^ and nationwide (39.6% of girls and 21.8% of boys).^12^ In a study conducted with European adolescents, only 10.3% of the sample had a self-rated health.^22^ Such differences in prevalence can be attributed to the socioeconomic, environmental, and cultural characteristics of the locality in which studies were conducted.^16^

The Human Development Index (HDI) of countries analyzed in the study by Granger et al.^22^ is very high, whereas that of Brazil is high.^23^ This factor might have influenced the self-perception health of the population. It is important to take into account the differences between Brazilian states with regard to development and social inequality; the northern and northeastern regions from Brazil have a medium HDI, whereas the southern regions have a high HDI.^24^

The prevalence of negative self-rated health was higher in girls than that in boys. This finding corroborates data compiled in a systematic review.^16^ It is known that girls are more judicious about their health. Given that girls have a better perception of physiological changes, they tend to identify less healthy habits and undergo routine examinations more frequently.^16^ It is understood that the more precise notion of health state that girls have may end up negatively influencing their self-rated health.

In the present study, no associations were observed between negative self-rated health and screen time in girls and boys. These findings diverge with the results of a meta-analysis, in which adolescents who spent more time in screen time were more prone to have poor self-rated health.^1^ Furthermore, excessive screen time is associated with physical, psychological, and social factors that negatively impact the health of children and adolescents,^6^ contributing to a more negative self-rated health.

In boys, an association was observed between physical inactivity and negative self-rated health. This result corroborates from previous studies with adolescents in Brazil^13^ and the United States.^25^ Here, we observed a high prevalence of physical inactivity in both sexes (boys: 89.7%; girls: 95.7%). Such a finding underscores the need to stimulate physical activity in this age group, given that adolescents who practice physical activity every day are eight times more likely to have a positive self-rated health compared with those who practice physical activity only once a week.^26^

The literature also shows a higher frequency of physically active boys compared to girls.^12^ Furthermore, physical activity is one of the main determinants for a positive self-perception of health.^26^ Thus, the association between negative self-perception of health and physical inactivity only in boys is possibly due to the fact that physical activity is a habitual behavior for them, and when there are reductions in physical activity levels, it ends up reflecting directly on these adolescents’ self-assessment of health.

In analyzing the combined effect of excessive screen time and physical inactivity, it was observed that boys with these two risk behaviors have a higher chance of having negative self-rated health compared with boys with only one risk factor. It is widely disseminated that low levels of screen time or regular physical activity alone can exert beneficial effects on self-rated health status.^1^ High levels of physical activity combined with low levels of screen time increased the probability of positive self-rated health by 1.37 times.^27^ Furthermore, there are associations between better self-rated physical and mental health and compliance with the proposed guidelines for physical activity and screen time, with even greater associations if it meets the recommendations for sleep duration.^21^ Although the present study analyzed the associations between physical inactivity and excessive screen time with negative self-rated health, it becomes possible, through the studies mentioned above, to analyze the dose-response effect of the combination of healthy behaviors on self-perception of health.

Population data showed that only 18% of girls and 38.5% of boys in Brazil are physically active^12^ and 70.9% of adolescents have excessive sedentary behaviors.^28^ Comparing the data of the present study with the results of previous studies, there is a similarity in the results regarding screen time; however, the number of adolescents meeting the global recommendations for physical activity is much lower. These findings indicate the need for interventions aimed at promoting a more active lifestyle, which may positively impact the self-rated health of adolescents. Self-rated health is a predictor of mortality^2^ and tends to remain unchanged or undergo little changes with advancing age.^29^ Furthermore, a negative self-rated health is associated with mental health problems^26^ and increased need to seek medical care due to psychological, musculoskeletal, gastrointestinal, and respiratory tract problems.^30^ Thus, positive self-rated health is highly important, as it has a great influence on the physical and psychological health of the population. Changing habits related to risk controls could lead to an improvement in health.

This study has some positive points and limitations. The representativeness of the sample is the strength of the study, allowing extrapolation of results to other populations of public school adolescents (14–18 years old). We also highlight that data on northern Brazil are scarce; thus, our results may be used for comparison in future studies on the self-perception health of Brazilian adolescents. The current study has an innovative nature for having analyzed combined associations between physical inactivity and excessive screen time with negative self-rated health; these factors, when analyzed separately, tend to be related. On the contrary, it should be noted that the design used here does not allow distinction between cause and effect regarding the study variables. Finally, the use of self-reported measures, which are widely applied in epidemiological studies because of their practicality and accessibility, may provide inaccurate data due to memory bias. Another factor to be considered is that the data were collected in 2011, which may have resulted in negative changes in the behavior of the evaluated public, especially after the isolation provided by the COVID-19 pandemic; therefore, attention should be paid to this information during the interpretation of the results. For future studies, it is suggested to continue the analysis of the combined effects that physical inactivity and excessive screen time have for negative self-rated health and to identify the impact that the pandemic had in relation to the data presented in this study. In addition, a longitudinal follow-up of the samples is suggested in order to understand how possible changes in risk behaviors will affect the adolescents’ self-rated health.

Physical inactivity and the combination of physical inactivity and sedentary behavior are exposure factors to negative self-rated health in adolescent boys, underscoring that this population group requires special attention. It is necessary to provide information on the benefits of physical activity and healthy behaviors through health-oriented educational interventions in schools aimed at adolescents and to conduct awareness campaigns for parents.
