# ERRATUM

**DOI:** 10.1590/1984-0462/2023/41/2022077erratum

**Published:** 2023-07-17

In the manuscript “Isolated and combined association of excessive screen time and physical inactivity with negative self-rated health in adolescents”, DOI: 10.1590/1984-0462/2023/41/2022077, published in the Rev Paul Pediatr. 2023;41:e2022077:

**On the top of the first page:**

Where it reads:

Jean Carlos Parmigiani de Marco

It should read:

Jean Carlos Parmigiani De Marco

